# Finding T-Cure: A CEO's vision for the future of adoptive cell immunotherapy

**DOI:** 10.4103/2666-2345.275504

**Published:** 2020-03-31

Gang Zeng, CEO and founder of emerging biotech T-Cure Bioscience has been at the forefront of T cell receptor (TCR)-based therapy research since his postdoctoral fellowship at the NCI, where he studied under TCR pioneer Steven A. Rosenberg.

5 years at the NCI, 17 years' professorship at UCLA and 61 publications later, Zeng has assembled a TCR dream-team to join him on his latest venture; T-Cure Biosciences Inc. Their mission statement? To deliver effective and durable cancer therapies by equipping patients with TCRs that readily target cancer cells.

Gang Zeng will be presenting his work at the **TCR-Based Therapies Summit (April 20–22, 2020) in Boston**, joining industry experts from Ziopharm Oncology, TCR2, Medigene, TScan Therapeutics, and many more. The TCR-Based Therapies Summit is the first ever meeting dedicated to target optimization, establishing comprehensive immune responses and directing T cells into the hostile tumor microenvironment, to accelerate safe and efficacious TCR-based therapies into the clinic. Today, Zeng shares his thoughts on the exploding TCR space.

*The Journal of Immunotherapy and Precision Oncology is proud to support the TCR-Based Therapies Summit and encourages readers to attend.*

***Hill:** What would you say sets T-Cure apart?*

**Zeng:** Our advantage is that we know the field very well. I am aware of the failures and the successes in the last 20 years. At T-Cure, we have developed a platform technology called iSORT that isolate TCRs against a wider range of antigens, not only NY-ESO-1, which could be considered a safe target, the platform can also isolate TCRs against a large variety of targets. For each antigen we have developed a suite that contains TCRs against a specific Antigen restricted to multiple HLA types, both class I and class II. Therefore, providing the patient presents that antigen, most likely we're able to deliver the TCR therapy to the patient.

***Hill:** Most TCR-based therapies to date have been developed using HLA class I and there is significantly less understanding of how to harness class II HLAs. What is the primary difference between these classes and how do each fit into the therapeutic landscape?*

**Zeng:** I like to see it as Class I are serving as a foot-soldier in a battle against cancer. They directly kill cancer cells. Class II TCR routes are more comprehensive, I like to consider them the commanders in this battle. The Class II T cells may play regulatory roles, they alter the tumor microenvironment, they can also enhance cross-priming in the tumor microenvironment. Class II influences the entire T cell repertoire including developing memory T cells. As a result, class II TCRs may direct the whole battlefield, even when the direct killing effect is less powerful than class I TCRs.

For some solid tumors, for example ovarian cancers, there is a huge regulatory T cell response and a lot of infiltrating T cells around the tumor. For this type of tumors, we would prefer to use class II. This would be a stage and tumor-dependent approach to maximize therapy. T-Cure is currently a start-up company and we are building up a class I and class II TCR suites around many novel Antigens. Next year, we aim to move the TCR suite into the clinic and patients will be carefully selected for the combinations and indication.

***Hill:** Starting up a company in the cell therapy space is no mean feat. Have you experienced particular challenges or successes in founding a cell therapy company?*

**Zeng:** Right, the challenge is enormous in all aspects. Luckily, we have a great team – small – but comprehensive in the fields of early discovery, translational research, clinical trials, regulations and R&D filing for TCRs. We also have industry expert, Dr. Gordon Parry who has great experience in developing therapies in many fields, including oncology. So, it has helped us to move from the bench to the clinic.

***Hill:** Has the growth of CAR-T therapy impacted TCR therapy development, and how?*

**Zeng:** I think the success of CAR-T has made people believe in cell therapy. Before CAR-T, cell therapy was kind of an obscure science. Now, people believe in CAR-T and TCR and the time to deliver TCR is just around the corner. In essence CAR-T is an antibody engineered onto a T cell. The monoclonal antibody technology was initiated in the mid-70's, developed and became a major drug in the mid 90's, some 20 years later. I think today is like 1994–1995 for the antibody field. I think we're a couple of years away from having TCR drugs on the market.

When you look at our immune system, it eliminates cancer and virus-infected cells by TCR. We don't have CARs in our bodies, and we don't use antibodies to eliminate cancers, we use TCRs, especially in solid tumors. So, what we are doing today is identifying the most powerful TCRs and engineering the TCR to a patient to mimic what happens in nature with clinical responders. I have no doubt that this is going to be a very powerful field.

***Hill:** I agree! This is why we are so excited to be holding the TCR-based summit in 2020. I think we will hear a lot of interesting conversations, just like this one, in April.*

**Zeng:** Yes, we'll look forward to that!

